# Excessive daytime napping independently associated with decreased insulin sensitivity in cross-sectional study – Hyogo Sleep Cardio-Autonomic Atherosclerosis cohort study

**DOI:** 10.3389/fendo.2023.1211705

**Published:** 2023-11-03

**Authors:** Miki Kakutani-Hatayama, Manabu Kadoya, Akiko Morimoto, Akio Miyoshi, Kae Kosaka-Hamamoto, Akinori Kanzaki, Kosuke Konishi, Yoshiki Kusunoki, Takuhito Syoji, Hidenori Koyama

**Affiliations:** Department of Diabetes, Endocrinology and Clinical Immunology, School of Medicine, Hyogo Medical University, Nishinomiya, Hyogo, Japan

**Keywords:** daytime nap, insulin sensitivity, actigraphy, heart rate variability, glucose intolerance

## Abstract

**Background:**

Although excessive daytime napping has been shown to be involved in diabetes occurrence, its impact on insulin secretion and sensitivity has not been elucidated. It is speculated that excessive napping disrupts the sleep-wake rhythm and increases sympathetic nerve activity during the day, resulting in decreased insulin sensitivity, which may be a mechanism leading to development of diabetes. We previously conducted a cross-sectional study that showed an association of autonomic dysfunction with decreased insulin sensitivity, though involvement of autonomic function in the association between napping and insulin sensitivity remained unclear. Furthermore, the effects of napping used to supplement to short nighttime sleep on insulin secretion and sensitivity are also unknown. In the present cross-sectional study, we examined the relationships of daytime nap duration and autonomic function with insulin secretion and sensitivity in 436 subjects enrolled in the Hyogo Sleep Cardio-Autonomic Atherosclerosis (HSCAA) Cohort Study who underwent a 75-g oral glucose tolerance test (75-g OGTT), after excluding those already diagnosed with diabetes.

**Methods:**

Daytime nap duration was objectively measured using actigraphy, with the subjects divided into the short (≤1 hour) and long (>1 hour) nap groups. Insulin secretion and sensitivity were determined using 75-g OGTT findings. Standard deviation of normal to normal R-R interval (SDNN), a measure of autonomic function, was also determined based on heart rate variability. Subgroup analysis was performed for the associations of napping with insulin secretion and sensitivity, with the results stratified by nighttime sleep duration of less or greater than six hours.

**Results:**

Subjects in the long nap group exhibited lower insulin sensitivity parameters (QUICKI: β=-0.135, p<0.01; Matsuda index: β=-0.119, p<0.05) independent of other clinical factors. In contrast, no associations with insulin secretion were found in either group. Furthermore, the association of long nap duration with insulin sensitivity was not confounded by SDNN. Specific subgroup analyses revealed more prominent associations of long nap habit with lower insulin sensitivity in subjects with a short nighttime sleep time (β=-0.137, p<0.05).

**Conclusion:**

Long daytime nap duration may be a potential risk factor for decreased insulin sensitivity.

## Introduction

Diabetes mellitus is a multifactorial disease caused by multiple genetic factors leading to decreased insulin secretion and sensitivity, in addition to lifestyle factors such as overeating and lack of exercise (1, 2). In recent years, attention has focused on the central nervous system, including the hypothalamus, brainstem, and dopamine reward system, which are involved in sleep and the autonomic nervous system, and have effects on insulin secretion and cardiovascular function shown in pancreatic β cells (3–7). Clinically, heart rate variability coefficient, an indicator of autonomic function, is a predictive factor for development of diabetes (8), while sleep duration and sleep quality, assessed by questionnaire, were also found to be associated with its development (9–11). Although nighttime sleep duration is associated with obesity, there are reports that fragmented sleep due to excessive daytime napping is more associated with obesity (12), drawing attention to the health effects of napping as well as nighttime sleep.

Previous studies have shown that an appropriate daytime napping habit improves work performance and mental health (13–15). On the other hand, excessive napping increased health risks such as the mental effects and cardiovascular disease have also been reported (16–20). As for the relationship with glucose metabolism, excessive daytime napping has been implicated in development of diabetes in a number of reported cases. A meta-analysis of 10 studies (four cross-sectional and six longitudinal cohort studies) found that napping for longer than one hour per day was associated with both the prevalence and incidence of diabetes, with a 31% increased risk of developing diabetes during the follow-up period. In contrast, no such association was found for those who took short naps of less than one hour (21). A cohort study of 2,620 subjects aged 60 years and older also found that those who took longer daytime naps (more than one hour per day) had a higher risk of developing diabetes compared with those who did not nap (22). It is speculated that excessive napping disrupts the sleep-wake rhythm (23) and increases sympathetic nerve activity during the day, resulting in decreased insulin sensitivity, which may be a mechanism leading to development of diabetes (10, 24). However, though some studies have noted that naps of 30 minutes or more significantly increase insulin resistance as compared to individuals who do not nap (25), others have found no clinically significant differences (26), thus the effects of daytime naps on insulin secretion and sensitivity remain unclear.

Autonomic dysfunction has been shown to occur even in the pre-diabetic stage (27–29), suggesting its possible contribution to development of diabetes. Furthermore, we previously reported findings of a cross-sectional study indicating that autonomic dysfunction is associated with decreased insulin sensitivity (30). For determining cardiac modulation, heart rate variability (HRV) is commonly used as a non-invasive procedure, as it is known to be a versatile marker of autonomic function (31, 32) and has been shown to be lower in patients with diabetes (8). Nevertheless, the mutual impact of nap duration and autonomic nervous function on insulin secretion and sensitivity in the pre-diabetic phase remains unresolved.

The Hyogo Sleep Cardio-Autonomic Atherosclerosis (HSCAA) cohort study was initiated in October 2010 with the aim to analyze quantitative changes in sleep duration, sleep quality, and apnea, including daytime napping, and their impact on autonomic function on metabolism and atherosclerosis. Based on quantitative assessments of nap time and autonomic function in 436 subjects who agreed to undergo a 75-g oral glucose tolerance test (75-g OGTT), and examinations of their interrelationship with insulin secretion and sensitivity, the present study aimed to explore the possibility that nap time and autonomic function are related to diabetes onset, whether napping as a supplement to short nighttime sleep is associated with insulin secretion and sensitivity, thus providing important information to help with early intervention for its prevention.

## Materials and methods

### Study population

**Figure 1:**
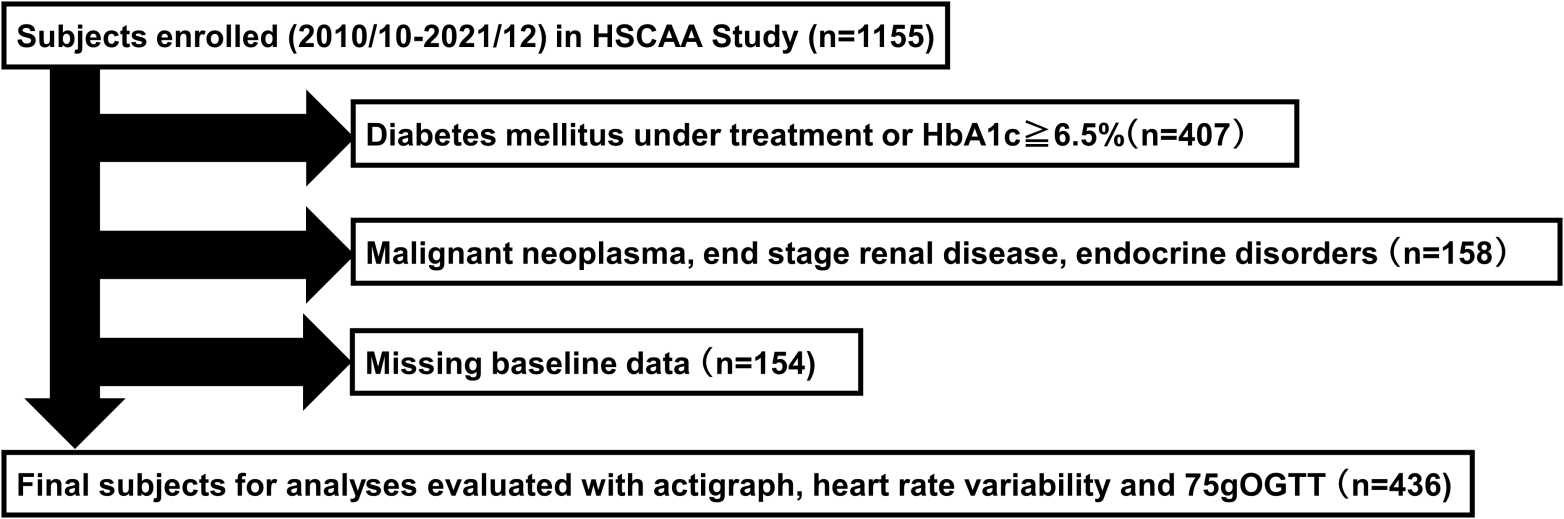
Flow of subject selection. HSCAA, Hyogo Sleep Cardio-Autonomic Atherosclerosis.

All subjects gave written informed consent to participate in the study according to a protocol approved by the Ethics Committee of Hyogo College of Medicine (approval no. 2351). The HSCAA study enrolled patients under treatment at the Department of Diabetes, Endocrinology and Metabolism, Hyogo Medical University Hospital (Hyogo, Japan) who had at least one cardiovascular risk factor, such as diabetes, hypertension, dyslipidemia, chronic kidney disease, history of cardiovascular events, obesity, or smoking habit, as well as sleep or autonomic nervous system function, or glucose metabolism disturbance (33, 34). The present cohort study aimed to investigate the relationship of sleep with autonomic function and glucose metabolism, metabolic syndrome, and atherosclerosis. This cross-sectional study was conducted as part of the HSCAA study and did not include patients taking sleeping pills. There were 1155 patients between the ages of 20 and 90 years enrolled in the HSCAA Study from October 2010 to December 2021. Those diagnosed with and treated for diabetes mellitus or HbA1c >6.5%, malignant neoplasms, overt endocrine disease, or renal failure were excluded, while there were also 154 missing baseline data, including monitor malfunctions. Thus, 436 patients able to undergo actigraph, active tracer, and 75-g OGTT examinations were included in this study (**Figure 1**).

### Assessment of classic cardiovascular risk factors

The medical history of each subject was obtained by interview to determine self-reported smoking and drinking habits, and history of cardiovascular events. Drinker was defined as consumption of alcoholic drinks at least three times a week and an individual with a current smoking habit as smoker. Body height and weight were measured, and body mass index (BMI) was calculated as weight (kilograms) divided by the square of height (meters) (kg/m^2^). Hypertension was defined as medical treatment for hypertension or systolic blood pressure ≥140 mmHg or diastolic blood pressure ≥90 mmHg. Dyslipidemia was defined as currently receiving treatment for dyslipidemia or elevated low-density lipoprotein cholesterol (≥140 mg/dl), decreased high-density lipoprotein cholesterol (<40 mg/dl), or elevated triglyceride (≥150 mg/dl) level (26). Blood samples were collected in the early morning following an overnight fast and quickly centrifuged to obtain plasma. Whole blood was used for hemoglobin A1c, EDTA plasma for glucose and lipids, and serum for other biochemical measurements.

### Biochemical parameters, 75-g OGTT, and calculation

For blood sample examinations, whole blood was used to measure hemoglobin A1c, and in EDTA-plasma was used for glucose (Glu) and insulin (IRI). Glucose was measured with a glucose oxidase method and insulin a radio immunometric assay (RIA-BEAD II; Dinabot, Tokyo, Japan). The patients fasted for at least 12 hours after dinner the previous day, then plasma samples for glucose (mmol/L) and insulin (pmol/L) measurements were collected at 0, 30, 60, 90, and 120 minutes after glucose loading, and 75-g OGTT was performed. The results were used to classify abnormal blood glucose levels [normal glucose tolerance (NGT), impaired glucose tolerance (IGT), diabetic type], and assess insulin secretion and sensitivity. Patients with fasting a plasma glucose level ≤109 mg/dl or two-hour plasma glucose ≤139 mg/dl were classified as NGT, fasting plasma glucose ≥110 mg/dl or two-hour plasma glucose from 140 to 199 mg/dl as IGT, and two-hour plasma glucose ≥200 mg/dl as DMtype. For estimation of insulin secretion and sensitivity, the following indices were calculated. Secretion: insulinogenic index = (IRI30 - IRI0)/(Glu30 - FPG) (35), corrected insulin response (CIR) = [(100 × IRI30)/(Glu30) × (Glu30 - 3.89)], disposition index = insulinogenic index × quantitative insulin sensitivity check index (QUICKI). Sensitivity: QUICKI = 1/(log IRI0 + log FPG) (36), BIGTT - sensitivity index (S_I_) = exp[4.9 - (0.00402 × IRI0) - (0.000556 × IRI30) - (0.00127 × IRI120) - (0.152 × FPG) - (0.00871 × Glu30) - (0.0373 × Glu120) - (0.145 × gender) - (0.0376 × BMI)] (37), Matsuda index = 10,000/[sqrt (FPG × IRI0 × mean PG × mean IRI)] (38).

### Determination of sleep conditions

Based on recent advances in technology, actigraphy is commonly used for diagnosis of sleep problems and findings demonstrating its effectiveness have been presented (30). Because a polysomnography device is complicated to fit and examinations with it are difficult to perform with a large number of subjects, the present study used findings for quantitative analyses of sleep duration and sleep quality obtained with an actigraph device (Ambulatory Monitoring, Inc., Ardley, NY, USA) (24, 32). The actigraph was attached to the wrist of the non-dominant arm and used twice for 24 consecutive hours. An actigraph converts signals generated by an accelerometer and collects them with frequency noted in hertz, then sums the values according to a user-specified time sampling interval termed an epoch and records them as activity counts. The approximate cutoff values for activity count are 0 to 1.5 for sedentary, 1.5 to 3 for light physical activity, 3 to 6 for moderate physical activity, and 6 or more for vigorous physical activity (39). Nap time was calculated as the sum of sleep time during waking hours. Wake-up time was set as the time when continuous activity was shown to be initiated on the actigraph. As used in several previous studies, we set a threshold of 1 hour for nap time, which has been shown to be associated with the risk of developing diabetes (21, 22, 40, 41), napping time was used to divide the patients into two groups; short (≤1 hour) and long (>1 hour). The activity mean is the average of the activity counts detected by the accelerometer during one minute, and the activity index is the percentage of the number of epochs where the activity count is greater than zero over the length of the measurement time period. Nocturnal activity index is a measure of sleep quality and calculated as movement of the whole body during sleep hours, with higher values indicating lower sleep quality (32, 33). Apnea-hypopnea index (AHI) was determined using an apnomonitor (SAS-2100, Teijin, Tokyo, Japan) (34).

### Assessment of autonomic nervous function

For assessment of cardiac autonomic function, HRV was measured using an active tracer device (AC-301A^®^, Arm Electronics, Tokyo, Japan) and the MemCalc Chiram 3 system, version 2.0 (Suwa Trust, Tokyo, Japan), with the standard deviation of the NN (RR) interval (SDNN), noted in previous reports as a noninvasive method (24, 25, 34–36), used as an index. Subjects wore an actigraph twice for 24 consecutive hours.

### Statistical analysis

Non-repeated t-tests and chi-square tests (categorical variables) were utilized to compare variables, including insulin secretion and sensitivity, between the short and long nap groups, as appropriate. Multiple regression analysis was performed to determine whether there was an independent relationship between the variables considered. Associations of age (under and over 50 years), gender, BMI (under and over 25 kg/m2), current smoker, alcohol consumption, hypertension, dyslipidemia, and HbA1c (under and over 5.8%) with disposition index, QUICKI, and Matsuda index were analyzed. For multivariate linear regression analyses, the covariates included age, gender, body mass index, current smoker, alcohol consumption, hypertension, dyslipidemia, and HbA1c. Multivariate linear regression analyses of factors associated with disposition index were performed using QUICKI and Matsuda index, with Model 1 including nap duration, Model 2 nocturnal sleep duration, Model 3 SDNN, Model 4 nap duration and nocturnal sleep duration, Model 5 nap duration and SDNN, and Model 6 nap duration, nocturnal sleep duration, and SDNN, in addition to the other covariates previously noted. Naptime and daytime physical activity were not included in the covariates, as simultaneous entry was deemed difficult due to multicollinearity.

The present patients were divided into two groups, those whose sleeping time was less than and more than six hours (22, 24), with subgroup analysis conducted to examine the relationship between Matsuda index, a measure of insulin sensitivity, and nap time. For multivariate linear regression analysis of factors associated with Matsuda index of patients with short and long nocturnal sleep duration, Model 1 included nap duration, Model 2 SDNN, and Model 3 nap duration and SDNN, in addition to the other covariates previously noted. Daytime nap time, nocturnal sleep time, insulin secretion/sensitivity index, and SDNN were natural log transformed (ln) to normalize the skewed distribution. All statistical analyses were performed using JMP^®^ Pro, version 15.2.0 (SAS Institute, Cary, NC, USA). The reported p values are two-tailed and were considered to be statistically significant at <0.05.

## Results

Comparisons of baseline characteristics for the subjects categorized by daytime napping duration are shown in **Table 1**. Those in the long nap group exhibited higher BMI and prevalence for alcohol consumption. In contrast, age, gender, BMI, HbA1c, AHI, and diabetes type shown by 75-g OGTT findings were not different between the groups. There are differences in clinical characteristics between people included in this analysis compared to those excluded (**Supplementary Table**).

**Table 1:** Comparisons of clinical characteristics categorized by daytime napping duration.

	Daytime napping duration		
	Short (≤1 hr)	Long (>1 hr)	P
Number of subjects	115	321	
Age, years	58.4	58.1	0.86
Male gender, n (%)	54 (47.0)	147 (45.8)	0.83
Body mass index, kg/m^2^	22.9±0.5	24.4±0.3	<0.01
Current smoker, n (%)	26 (22.6)	86 (26.8)	0.38
Alcohol consumption, n (%)	51 (44.4)	106 (33.0)	0.03
Hypertension, n (%)	70 (60.9)	193 (60.1)	0.89
Dyslipidemia, n (%)	60 (52.2)	172 (53.6)	0.80
HbA1c, %	5.7±0.0	5.7±0.0	0.46
OGTT			
NGT, n (%)	36 (38.7)	91 (36.3)	0.68
IGT, n (%)	40 (43.0)	114 (45.4)	0.69
DM Type, n (%)	17 (18.3)	46 (18.3)	0.99
Nap duration (min)	34.1±9.9	189.0±5.9	<0.01
Nocturnal sleep duration (min)	362.3±10.6	319.1±6.5	<0.01
Nocturnal activity index	34.52±1.61	33.18±0.97	0.48
Daytime activity mean	155.58±4.47	119.43±2.68	<0.01
AHI	8.6±1.0	9.4±0.6	0.46

Data are presented as the mean ± standard error (SE) for continuous variables, and number (%) for dichotomous variables. P values are shown for comparisons of mean values for the groups (unrepeated t-test) or percentages (chi-squared test). OGTT, oral glucose tolerance test; NGT, normal glucose tolerance; IGT, impaired glucose tolerance; DM, diabetes mellitus; AHI, apnea hypopnea index.

**Figure 2:**
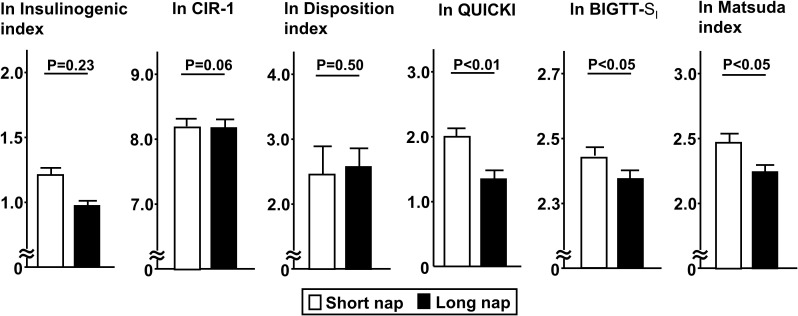
Comparisons of indices for insulin secretion and sensitivity between short and long nap groups. Parameters for insulin secretion [insulinogenic index, corrected insulin response (CIR), disposition index] and insulin sensitivity (QUICKI, BIGTT- S_I_, Matsuda index) were calculated based on plasma glucose and insulin levels determined at 0, 30, 60, 90, 120 minutes with a 75-g oral glucose tolerance test. These parameters were natural logarithm transformed to achieve a normal distribution. Each column shows the mean ± standard error. P values were determined using Student’s t-test.

**Figure 3:**
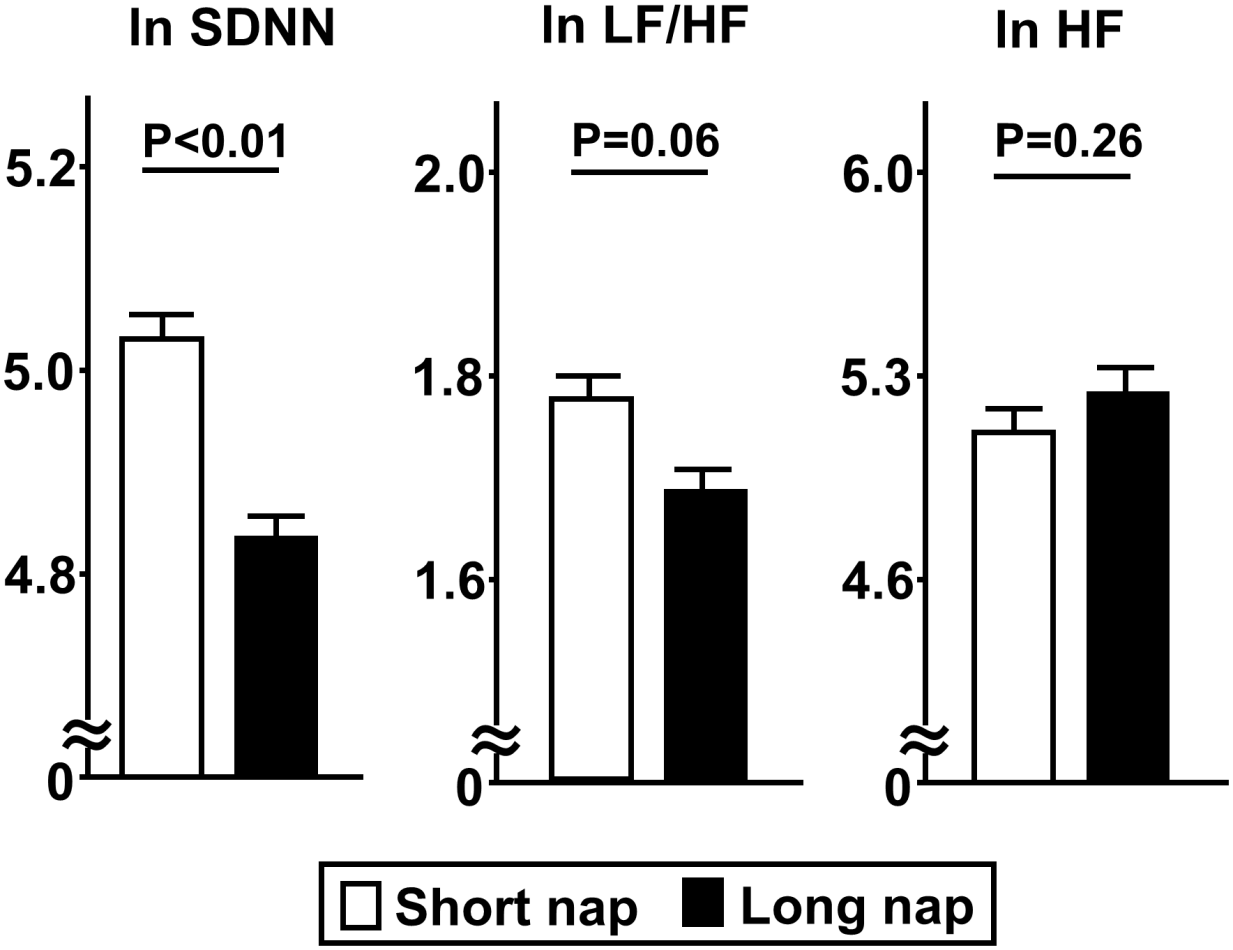
Comparisons of indices for HRV between short and long nap groups. As parameters for HRV, SDNN [standard deviation of NN (RR)), LF/HF (low-frequency domain/ high-frequency domain), and HF (high-frequency domain) were determined. These parameters were natural logarithm transformed to achieve a normal distribution. Each column shows the mean ± standard error. P values were determined using Student’s t-test.

**Figure 4:**
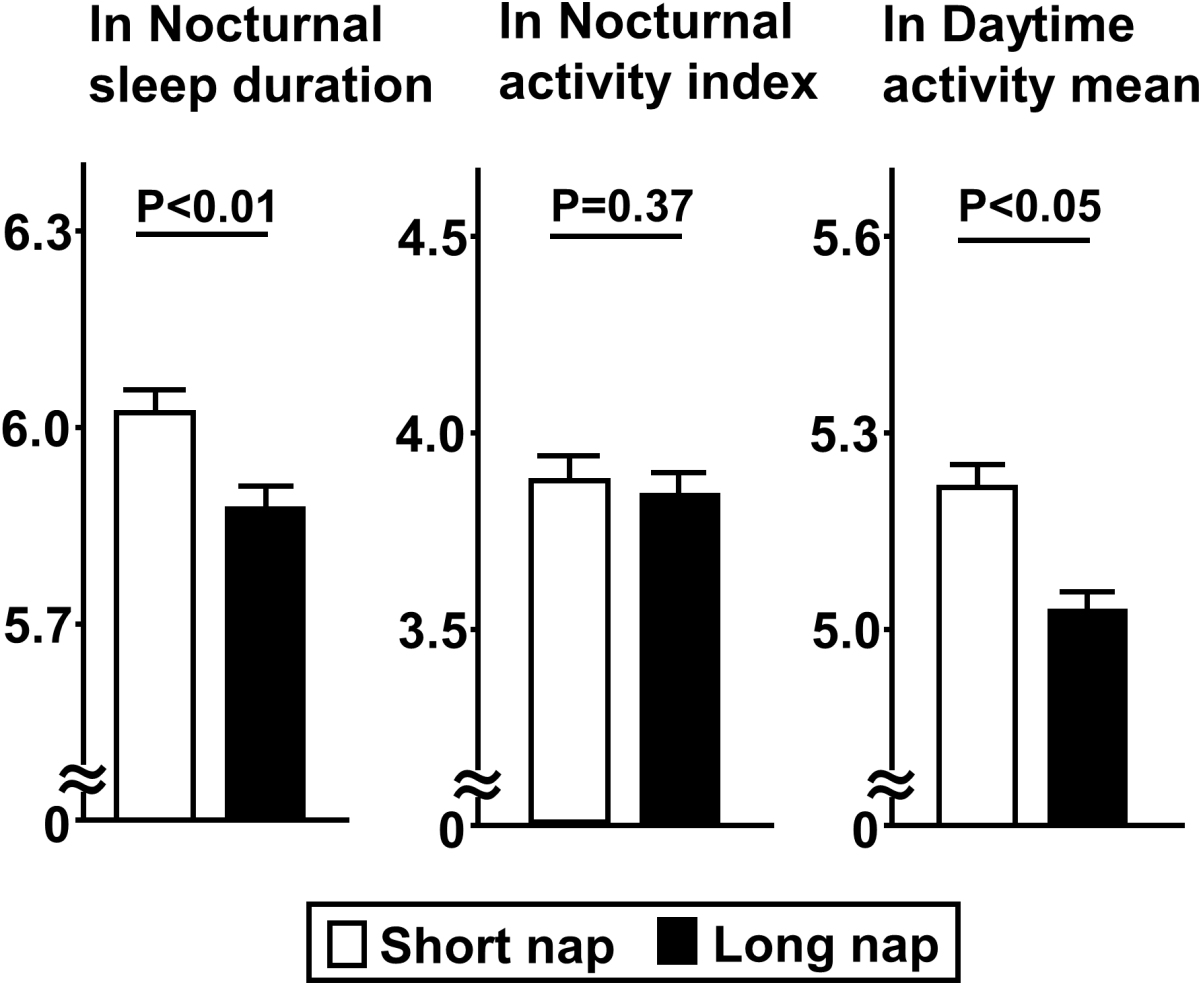
Comparisons of indices for sleep duration and quality between short and long nap groups. Parameters for sleep duration, quality, and daytime activity were determined. These parameters were natural logarithm transformed to achieve a normal distribution. Each column shows the mean ± standard error. P values were determined using Student’s t-test.

Comparisons of insulin secretion (insulinogenic index, CIR, disposition index) and sensitivity (QUICKI, BIGTT- S_I_, Matsuda index), calculated using a 75-g OGTT, between the groups are presented in **Figure 2**. Insulin sensitivity values, such as QUICKI (p<0.01), BIGTT- S_I_ (p<0.05), and Matsuda index (p<0.05), for the long nap group were lower, while parameters for insulin secretion were not different. For autonomic function, SDNN for the long nap group was lower (p<0.01), while LF/HF and HF were not different (**Figure 3**). Additionally, sleep duration was shorter (p<0.01) and daytime activity lower (p<0.05) for the long nap group, while nocturnal activity index was not different (**Figure 4**).

Next, the relationships of disposition index for insulin secretion, and QUICKI and Matsuda index for insulin sensitivity with other clinical factors were examined (**Table 2**). Disposition index values were lower for subjects with advanced age (>50 years), alcohol consumption, hypertension, and high HbA1c, while QUICKI values were lower for subjects with advanced age, high BMI, current smoking habit, dyslipidemia, and high HbA1c. On the other hand, Matsuda index values were higher for subjects with advanced age, high BMI, current smoking habit, and dyslipidemia.

**Table 2:** Analyses of factors associated with disposition index, QUICKI, and Matsuda index.

Variables	Disposition index		QUICKI		Matsuda index	
	Mean ± SE	P	Mean ± SE	P	Mean ± SE	P
Age						
≤50 years (n=132)	-2.20±0.09		-1.82±0.02		1.61±0.08	
>50 years (n=304)	-2.69±0.06	<0.01	-1.77±0.01	<0.05	1.92±0.05	<0.01
Gender						
male (n=201)	-2.63±0.07		-1.80±0.01		1.76±0.06	
female (n=235)	-2.45±0.07	0.06	-1.76±0.01	0.06	1.89±0.06	0.12
Body mass index						
<25 kg/m^2^ (n=284)	-2.56±0.06		-1.74±0.01		2.04±0.05	
≥25 kg/m^2^ (n=152)	-2.49±0.08	0.49	-1.86±0.01	<0.01	1.45±0.07	<0.01
Current smoker						
yes (n=112)	-2.62±0.10		-1.82±0.02		1.67±0.08	
no (n=324)	-2.50±0.06	0.31	-1.77±0.01	<0.05	1.88±0.05	<0.05
Alcohol consumption						
yes (n=157)	-2.75±0.08		-1.77±0.02		1.89±0.07	
no (n=279)	-2.42±0.06	<0.01	-1.79±0.01	0.43	1.79±0.06	0.30
Hypertension						
yes (n=263)	-2.70±0.06		-1.79±0.01		1.84±0.06	
no (n=173)	-2.30±0.08	<0.01	-1.78±0.01	0.63	1.81±0.07	0.81
Dyslipidemia						
yes (n=232)	-2.52±0.07		-1.82±0.01		1.66±0.06	
no (n=204)	-2.56±0.07	0.69	-1.74±0.01	<0.01	2.00±0.06	<0.01
HbA1c						
<5.8% (n=274)	2.32±0.07		-1.77±0.01		1.89±0.06	
≥5.8% (n=162)	-2.81±0.07	<0.01	-1.80±0.01	<0.01	1.75±0.07	0.11

Student’s t-test was used for categorical variables and the obtained data are presented as mean ± standard error (SE). SDNN, standard deviation of NN(RR) interval.

Multiple linear regression analysis was performed to further examine whether the associations of nap time, sleep duration, and SDNN with body mass index, QUICKI, and Matsuda index were independent of potential clinical confounders (**Table 3**). The adjusted R^2^ for models 1-6 were all between 0.13 and 0.26, which were considered reasonable as goodness of fit. Those results showed that long nap duration remained inversely associated with QUICKI (β=-0.135, p<0.01) and Matsuda index (β=-0.119, p<0.05) (Model 1), while SDNN showed a positive association with QUICKI (β=0.118, p<0.05) (Model 3). Furthermore, even after addition of sleep duration (Model 4), SDNN (Model 5), as well as both (Model 6) to Model 1, long nap duration remained inversely associated with QUICKI (β=-0.127, p<0.05) and Matsuda index (β=-0.123, p<0.05). On the other hand, nap duration was not associated with insulin secretion or sensitivity (Model 2).

**Table 3:** Multivariate linear regression analysis of factors associated with disposition index, QUICKI, and Matsuda index.

Variables	Disposition index		QUICKI		Matsuda index	
	β (SE)	adjusted R^2^	β (SE)	adjusted R^2^	β (SE)	adjusted R^2^
Model 1		0.15		0.22		0.25
Nap duration (≤1 hr=0, >1 hr=1)	0.003 (0.05)		-0.135 (0.01)**		-0.119 (0.04)*	
Model 2		0.15		0.20		0.24
Nocturnal sleep duration	-0.009 (0.10)		-0.020 (0.02)		-0.043 (0.08)	
Model 3		0.14		0.19		0.23
SDNN	0.001 (0.17)		0.118 (0.03)*		0.093 (0.14)	
Model 4		0.15		0.21		0.26
Nap duration (≤1 hr=0, >1 hr=1)	0.001 (0.06)		-0.141 (0.01)**		-0.135 (0.05)*	
Nocturnal sleep duration	-0.009 (0.11)		-0.049 (0.02)		-0.072 (0.08)	
Model 5		0.14		0.21		0.24
Nap duration (≤1 hr=0, >1 hr=1)	0.007 (0.06)		-0.129 (0.01)*		-0.116 (0.05)*	
SDNN	0.002 (0.18)		0.098 (0.03)		0.075 (0.14)	
Model 6		0.13		0.20		0.25
Nap duration (≤1 hr=0, >1 hr=1)	0.009 (0.06)		-0.127 (0.01)*		-0.123 (0.05)*	
Nocturnal sleep duration	0.004 (0.11)		-0.024 (0.02)		-0.060 (0.09)	
SDNN	0.008 (0.18)		0.112 (0.03)*		0.099 (0.14)	

Standard beta coefficient and standard error (SE) values are shown. For multivariate linear regression analyses, the covariates included age, gender, body mass index, current smoker, alcohol consumption, hypertension, dyslipidemia, and HbA1c. Model 1 includes Nap duration, Model 2 Nocturnal sleep duration, Model 3 SDNN, Model 4 Nap duration and Nocturnal sleep duration, Model 5 Nap duration and SDNN, and Model 6 Nap duration, Nocturnal sleep duration and SDNN, in addition to other covariates already noted. SDNN, standard deviation of NN(RR) interval. *p<0.05, **p<0.01.

Finally, specific subgroup analyses showed more prominent associations of long nap duration with lower Matsuda index value in subjects with a short sleep duration (≤6 hours) (β=-0.137, p<0.05, adjusted R^2^=0.23), while that was not a factor in those with a long sleep duration (>6 hours) (β=-0.156, p=0.06, adjusted R^2^=0.27) (**Table 4**).

**Table 4:** Multivariate linear regression analysis of factors associated with Matsuda index in short- and long-time nocturnal sleep duration groups.

Variables	β (SE)	adjusted R^2^
Subjects with short sleep duration (≤6 hr) (n=255)		
Model 1		0.23
Nap duration (≤1 hr=0, >1 hr=1)	-0.137 (0.07)*	
Model 2		0.24
SDNN	0.117 (0.19)	
Model 3		0.25
Nap duration (≤1 hr=0, >1 hr=1)	-0.118 (0.07)	
SDNN	0.094 (0.19)	
Subjects with long sleep duration (>6 hr) (n=181)		
Model 1		0.27
Nap duration (≤1 hr=0, >1 hr=1)	-0.156 (0.06)	
Model 2		0.24
SDNN	0.122 (0.23)	
Model 3		0.26
Nap duration (≤1 hr=0, >1 hr=1)	-0.157 (0.06)	
SDNN	0.112 (0.23)	

Standard beta coefficient and standard error (SE) values are shown. For multivariate linear regression analyses, the covariates included age, gender, body mass index, current smoker, alcohol consumption, hypertension, dyslipidemia, and HbA1c. Model 1 includes Nap duration, Model 2 SDNN, and Model 3 Nap duration and SDNN, in addition to other covariates already noted. SDNN, standard deviation of NN(RR) interval. *p<0.05.

## Discussion

Results obtained in the present study showed that a nap time of greater than one hour was independently associated with decreased insulin sensitivity related to autonomic dysfunction. Moreover, specific subgroup analyses revealed a more prominent association of long nap time with lower insulin sensitivity in individuals with a nighttime sleep duration of six hours or less.

A previous report noted that shorter (six hours) and longer (eight hours) sleep durations were associated with incident diabetes mellitus (22). Additionally, recent studies have shown that excessive napping for more than one hour increases the risk for development of diabetes, in contrast to a shorter nap duration (21, 22, 40, 41). Previously reported ORs for diabetes onset were 1.2395% (CI 1.18 .1.29) for individuals who nap for one hour or less and 1.55 (95% CI 1.45.1.66) for those who nap for more than one hour, as compared to those who do not nap (40). This is considered to be due to decreased sleep duration and quality caused by a longer nap duration (42–44).

Another report speculated that long naps during the day may affect the sleep-wake cycle and noted that disruption of the circadian rhythm is an environmental risk factor predisposing to type 2 diabetes, as fragmented sleep induces sympathetic stimulation during the day, a mechanism possibly related to insulin sensitivity (24). Thus, misalignment of the circadian rhythm results in reduced insulin sensitivity due to sympathetic and other influences. Our previous study found that autonomic function is associated with decreased insulin sensitivity (30). Initially, we speculated that a prolonged nap duration would thus be shown to be associated with decreased insulin sensitivity via decreased autonomic function, and the present results showed that autonomic function was lower in the long as compared with the short group. However, multivariate analysis revealed that nap duration was independently associated with decreased insulin sensitivity. This may be because several factors other than autonomic function are involved in the association of long nap duration with development of diabetes.

Excessive napping may also decrease daytime activity and calorie consumption (45), leading to obesity (46), increased insulin resistance, and increased risk of developing diabetes. The present long nap group had lower daytime activity and higher BMI. Although nap time was associated with drinkers in this study, the results of multiple regression analysis with alcohol consumption as a covariate, which showed that excessive napping was independently associated with insulin sensitivity, did not mean that napping was associated with reduced insulin sensitivity via alcohol consumption. Although napping as a complement to short nighttime sleep may be refreshing, naps should total no more than one hour each day. Many previous reports have used self-reported questionnaires (Pittsburgh Sleep Quality Index) to assess sleep and nap duration (40, 47–49), while objectively obtained quantitative data based on actigraph results were used in the present investigation. Thus, the results may be different as compared to self-reported and polysomnographic assessments. It is conceivable that use of self-reported questionnaires indicate shorter nap durations, thus leading to underestimation. In a study in which polysomnography and actigraph were simultaneously used to assess nap duration, that determined by actigraph findings was correlated with polysomnography results (50). Thus, use of an actigraph to determine actual nap duration is considered to be reasonable.

Napping may mitigate adverse health effects in individuals who sleep for a short time at night. In the present study, excessive napping was associated with decreased insulin sensitivity only in subjects with less than six nighttime sleep hours, suggesting that a nap of one hour or more may contribute to increased risk of diabetes development, even when naps are used to compensate for short nighttime sleep. Another study found that daytime napping is a symptom of obstructive sleep apnea (51), which induces oxygen deprivation, and also causes elevated catecholamines and cortisol, resulting in increased insulin resistance and contributing to impaired glucose tolerance (40, 52). However, the present study found no difference in AHI between the long and short groups. This may be due to the fact that few of the subjects were affected by moderate or severe sleep apnea.

This study has several limitations. First, the results may not be generalizable for all patients with varying glycemic status. Second, due to the cross-sectional design, a causal relationship could not be determined. To avoid loss of statistical power, subjects who were first diagnosed as diabetic based on glucose tolerance testing were not excluded. Third, data for subgroups based on different levels of glucose intolerance (NGT, IGT, DM types) were not analyzed so as to avoid statistical power loss. Fourth, sleep assessment with actigraphy was performed during hospitalization, thus actual sleep behavior during daily life could not be assessed. The results during hospitalization may not be exactly the same as napping habits in daily life, but we believe that daily habits may be reflected in how patients spend their time during hospitalization. Fifth, polysomnography, considered as the gold standard for evaluating sleep, was not used in this study and the possibility that the results may differ from those obtained with that method cannot be ruled out. Finally, the present results did not reveal any potential mechanisms involved in the association of excessive napping with autonomic function or insulin sensitivity. Leptin is known to be involved in both sleep and autonomic function, and considered as a potential candidate factor for napping, autonomic function, and insulin sensitivity. In clinical studies, plasma leptin levels have been shown to be associated with insulin sensitivity in both healthy and obese women, as well as patients with type 2 diabetes. Nevertheless, the present study is the first to report an association of long nap duration with increased insulin sensitivity. We believe that these results provide important information to better understand the pathophysiological significance of napping as a factor related to abnormal blood glucose levels.

In conclusion, in patients without apparent diabetes, napping for a long period of time was found to be independently associated with decreased insulin sensitivity from autonomic dysfunction. Additionally, a prominent association of long nap duration with lower insulin sensitivity was observed in patients with a short nighttime sleep period.

## Data availability statement

The raw data supporting the conclusions of this article will be made available by the authors, without undue reservation.

## Ethics statement

The studies involving humans were approved by Department of Diabetes, Endocrinology and Clinical Immunology, Hyogo Medical University. The studies were conducted in accordance with the local legislation and institutional requirements. The participants provided their written informed consent to participate in this study.

## Author contributions

MK-H, MK and HK: conceptualization, methodology, software use. MK-H, MK, AMo, AMi, KK-H, AK: data curation. MK-H and MK: writing - original draft preparation. KK, YK, and TS: supervision. MK: validation. MK and HK: writing - reviewing and editing. All authors contributed to the article and approved the submitted version.
