# Supplementary material for: Excessive daytime napping independently associated with decreased insulin sensitivity in cross-sectional study – Hyogo Sleep Cardio-Autonomic Atherosclerosis cohort study
Source: Front Endocrinol (Lausanne). 2023 Nov 3;14:1211705. doi: 10.3389/fendo.2023.1211705 (PMC10656607; doi:10.3389/fendo.2023.1211705)
Supplement: Supplementary file 1 [file Table_1.docx]

| **Supplement table. Comparisons of clinical characteristics categorized by population included or excluded this analysis** | | | |
| --- | --- | --- | --- |
|  | Final subjects | Diabetes Mellitus | Malignant disease , end stage renal disease , endocrine disorders |
| Number of subjects | 436 | 407 | 158 |
| Age, years | 58.2±0.7 | 62.3±0.7* | 56.7±1.2 |
| Male gender, n (%) | 201 (46.1) | 202 (49.9) | 76 (48.1) |
| Body mass index, kg/m2 | 24.0±0.2 | 25.1±0.3* | 24.0±0.4 |
| Current smoker, n (%) | 112 (25.7) | 97 (29.0) | 32 (20.4) |
| Alcohol consumption, n (%) | 157 (36.0) | 132 (39.9) | 61 (39.4) |
| Hypertension, n (%) | 263 (60.3) | 256 (71.5)* | 112 (70.9)* |
| Dyslipidemia, n (%) | 232 (53.2) | 211 (63.2)* | 80 (51.0) |
| HbA1c, % | 5.7±0.0 | 7.9±0.1* | 5.4±0.0* |
| Data are presented as the mean ± standard error (SE) for continuous variables, and number (%) for dichotomous variables. P values are shown for comparisons of mean values for the groups (unrepeated t-test) or percentages (chi-squared test). *p<0.05 | | | |
